# Clostridial Prosthetic Joint Infections: A Review of the Literature, Management Strategies, and Outcomes

**DOI:** 10.7759/cureus.97437

**Published:** 2025-11-21

**Authors:** Dimitris Panayiotou, Stuart Drazich-Taylor, Lee X Ting, Abid Hussain

**Affiliations:** 1 Trauma and Orthopaedics, University Hospitals Birmingham NHS Foundation Trust, Birmingham, GBR; 2 Microbiology, University Hospitals Birmingham NHS Foundation Trust, Birmingham, GBR; 3 Infectious Disease, University Hospitals Birmingham NHS Foundation Trust, Birmingham, GBR

**Keywords:** anaerobe, clostridioides difficile, clostridium bifermentans, clostridium cadaveris, clostridium disporicum, clostridium perfringens, clostridium ramosum, clostridium septicum, prosthetic joint infection (pji), review of literature

## Abstract

Clostridial prosthetic joint infections (PJIs) are rare causes of anaerobic implant infections, with limited published data to guide diagnosis or management. Most reported cases involve *Clostridium perfringens* or *C. difficile*, although other *Clostridium* species can also be implicated. Owing to their rarity and diverse clinical behaviour, the optimal management of clostridial PJIs remains uncertain. All published cases of clostridial PJIs were identified through a literature review up to September 2025. Cases were included if the prosthetic joint infection was clearly attributed to a *Clostridium *species and excluded if data were insufficient. Data was extracted on patient demographics, microbiology, management, and outcomes. Thirty-four cases were included: *C. perfringens* (n = 16), *C. difficile* (n = 11), and other *Clostridium* species (n = 7). The majority of patients were older adults with significant comorbidity or immune suppression. *C. perfringens* infections were predominantly early-onset, often associated with gas formation and favourable outcomes after surgical intervention and penicillin-based therapy. *C. difficile* PJIs were usually monomicrobial, often treated with metronidazole, while other *Clostridium* species presented as late-onset infections, frequently in association with malignancy or immunosuppression. Antimicrobial therapy varied between cases, reflecting the absence of standardised regimens and the need for individualised treatment approaches. Across all reported cases, the majority achieved infection control following combined surgical and antimicrobial therapy. This review consolidates all available data on clostridial PJIs to date, defines their microbiological and clinical spectrum, and provides practical insight into the diagnosis and management of these uncommon infections while highlighting the need for greater awareness and standardised treatment strategies.

## Introduction and background

Prosthetic joint infections (PJIs) are a serious complication of arthroplasty, with major consequences for both overall patient health and joint function. The incidence of PJI is estimated at 1-2% of primary arthroplasties [1-3]. With an ageing population, the need for arthroplasty continues to rise [4]; as a consequence, the absolute number of infections is likely to grow. PJIs carry substantial morbidity, healthcare costs, and a risk of treatment failure if not recognised and managed appropriately.

More than 50% of PJIs are caused by staphylococci [5]. However, anaerobic pathogens represent a meaningful minority and are under-detected when appropriate culture techniques are not used [6]. Reported proportions vary by joint, infection timing, and diagnostic methods, but anaerobes have been estimated to account for approximately 3-24% of PJIs [5].

*Clostridium* species represent a rare but clinically important subset of anaerobic PJIs, distinguished by their environmental persistence, toxin-mediated pathogenicity, and fastidious culture requirements [6]. They also pose diagnostic and therapeutic challenges and carry potential associations with systemic disease [6,7]. Reported cases are sparse, documented mainly as single-case reports or small series across several decades [8-10], limiting clinicians’ ability to recognise consistent patterns or anticipate outcomes. Management strategies vary widely, with ongoing uncertainty regarding the role of prosthesis retention, optimal antimicrobial duration, and the extent of investigation required for potential underlying conditions such as gastrointestinal disease or malignancy [11,12].

Diagnosis of PJI remains challenging, and the absence of universally accepted diagnostic criteria for PJI has been repeatedly highlighted in the literature [4,5]. The Musculoskeletal Infection Society (MSIS) first established consensus criteria in 2011, later refined by the International Consensus Meeting (ICM) in 2018 to include a points-based algorithm [13,14]. The Infectious Diseases Society of America (IDSA) published complementary guidance integrating microbiological and clinical aspects [1]. The European Bone and Joint Infection Society (EBJIS) has recently proposed a three-level diagnostic framework categorising cases as infection unlikely, likely, or confirmed. This framework incorporates clinical findings, serological inflammatory markers, synovial fluid cytology, synovial biomarker analysis, microbiological cultures, and histopathology, with the aim of improving diagnostic standardisation and comparability across centres [15].

Diagnostic accuracy in PJIs has improved markedly with the introduction of advanced microbiological methods such as matrix-assisted laser desorption/ionisation time-of-flight (MALDI-TOF) mass spectrometry and 16S ribosomal RNA (rRNA) gene sequencing, which enable rapid and reliable identification of fastidious or culture-negative organisms [16,17]. However, significant variability in laboratory practice persists, with a recent global survey highlighting heterogeneity in sampling protocols, anaerobic culture use, and identification techniques across centres [18]. These differences continue to influence the detection and reporting of anaerobic pathogens, including *Clostridium* species.

The management of PJI combines surgical and medical approaches. The main surgical strategies include debridement, antibiotics, and implant retention (DAIR) and exchange arthroplasty. DAIR involves the removal of infected tissue while preserving the prosthesis. Exchange arthroplasty can be performed as a one-stage procedure, which involves immediate reimplantation of a new prosthesis, or as a two-stage procedure, involving prosthesis removal, placement of an antibiotic-loaded spacer, followed by delayed reimplantation of the prosthesis after antibiotic therapy. Arthrodesis, amputation, chronic antibiotic suppression, or arthroplasty resection without reimplantation are management strategies reserved for complex cases [5]. Guidelines such as those published by the IDSA provide overarching principles to standardise management and refine clinical practice, but they do not specifically address infections caused by *Clostridium* species [1].

Antimicrobial therapy remains a cornerstone of PJI management, complementing surgical intervention. Treatment traditionally involves an initial intravenous phase followed by prolonged oral suppression, with total duration varying according to surgical strategy and organism [1,4]. Recent evidence from a large multicentre randomised controlled trial demonstrated that early transition to oral antibiotics can be as effective as prolonged intravenous courses [19]. Antimicrobial selection must account for biofilm-associated resistance, tissue penetration, and patient comorbidities [4]. However, evidence guiding antimicrobial regimens for anaerobic or toxin-producing species, including clostridia, remains limited, and reported management is largely empirical.

Our review aims to summarise published cases of clostridial PJI to describe their clinical features, management, and outcomes. It seeks to characterise demographic and clinical presentation patterns, including infection timing and probable sources of bacteraemia, while also summarising surgical and antimicrobial strategies and their reported outcomes. Given that available evidence is limited to individual case reports and small series, we undertook a narrative synthesis to consolidate these observations and provide practical insights to support clinical recognition and management of these rare but clinically significant infections.

## Review

Methods

Search Strategy

A PubMed search identified case reports and series of clostridial PJIs published up to 30 September 2025. The following search string was applied across all fields: (((prosthetic joint infection) OR (PJI)) OR (periprosthetic joint infection)) AND (((clostridium) OR (clostridial)) OR (clostridioides)). This search yielded 66 results, of which 24 articles met the inclusion criteria. An additional eight studies were identified through manual reference list screening of included articles. Three case series were excluded as they did not provide patient-level demographic or clinical data specific to the prosthetic joint infection cases [20-22].

Eligibility Criteria

Reports were included if a *Clostridium* species was isolated from a prosthetic joint and sufficient clinical and microbiological data were available, including patient demographics, infection timing, joint involved, organism identification, and details of surgical or antimicrobial management. Articles were excluded if they were not published in English, formed part of a larger series without distinct prosthetic joint infection data, or lacked sufficient clinical or microbiological detail for analysis.

Data Extraction and Analysis

Eligible cases were entered into structured tables and grouped as *Clostridium perfringens*, *Clostridioides difficile* (formerly *Clostridium difficile*), or other *Clostridium* species for comparative analysis. Other *Clostridium* species were grouped together owing to their rarity, with the specific organism for each case identified in the corresponding table to maintain species-level transparency. For each case, data were extracted on patient demographics, affected joint, infection timing, causative organism, surgical management, antibiotic therapy, and outcomes, and were organised into summary tables with descriptive statistics calculated manually. Infection timing was categorised as early (within three months of surgery) or late (more than three months after surgery) in line with commonly used classification schemes in the PJI literature [1]. Where available, details regarding antibiotic route, dose, and duration, as well as blood culture results, immunosuppressive conditions, gastrointestinal symptoms, and radiological evidence of gas were also recorded. As this was a narrative review synthesising descriptive case reports and small series, a formal risk-of-bias assessment and Preferred Reporting Items for Systematic Reviews and Meta-Analyses (PRISMA) framework were not applied.

Clostridium perfringens

*C. perfringens* is a gram-positive, anaerobic, spore-forming bacillus. It is found in soil and the gastrointestinal tracts of humans and animals, and it commonly colonises wounds or necrotic tissue. It is classified into toxinotypes (A-G) based on the toxins it produces [23], with Type A dominating in human infections, responsible for both gas gangrene and food poisoning. In total, 16 cases of *C. perfringens* PJIs were identified in the literature, and are summarised in Table 1.

**Table 1:** Summary of Clostridium perfringens PJIs M: male, F: female, IV: intravenous, PJI: prosthetic joint infection, OD: once daily, BD: twice daily, TDS: three times daily, QDS: four times daily, N/A: data not available, Early infection: occurred within three months of surgery, Late infection: occurred more than three months after surgery, HIV: human immunodeficiency virus, DAIR: debridement, antibiotics, and implant retention, *C. perfringens*: *Clostridium perfringens*

Author(s)	Year	Joint	Age	Sex	Early/Late Infection	Type of infection	Blood cultures	Immunosuppressive conditions	Gastrointestinal symptoms	Radiological evidence of intra-articular or periarticular gas	Surgery	Antibiotics (after culture results)	Outcome
Rush JH [24]	1976	Hip	62	M	Early	Monomicrobial	N/A	No	No	Yes	DAIR	Penicillin 5 million units QDS, cloxacillin 500 mg QDS	Secondary infection with *Pseudomonas pyocyanea*, sinus tract development with persistent discharge, sinus exploration eight months later, further persistent sinus developed eight months after this (persistent discharge)
Rush JH [24]	1976	Hip	66	F	Early	Monomicrobial	N/A	N/A	No	N/A	No surgery	Penicillin and cloxacillin 500 mg QDS	No recurrence at one and a half years
Maniloff G, et al. [25]	1987	Knee	62	F	Late	Monomicrobial	No blood cultures at time of PJI, blood cultures positive for *C. perfringens* 10 months before PJI	Rheumatoid arthritis treated with systemic steroids	No GI symptoms at time of PJI, abdominal pain, stool incontinence and biliary stones 10 months prior to PJI	N/A	DAIR	IV penicillin for 26 days then oral penicillin for six months	No recurrence at 17 months
Wilde AH, et al. [26]	1988	Knee	64	M	Early	Monomicrobial, developed secondary infection (*Enterobacter cloacae*)	Positive for *C. perfringens*	No	Ascending cholangitis on eighth postoperative day following primary arthroplasty	Yes	Implant removal, two debridements, reimplantation of arthroplasty followed by removal and arthrodesis (due to *Enterobacter cloacae *infection after reimplantation)	IV penicillin 3 million units every four hours for three weeks	No recurrence at 24 months
Stern SH, Sculco TP [27]	1988	Knee	66	F	Early	Monomicrobial	N/A	No	No	N/A	Two-stage exchange	IV penicillin six weeks	No recurrence at two and a half years
Kibbler CC, et al. [28]	1991	Hip	77	F	Late	Monomicrobial	Positive for *C. perfringens*	History of ovarian carcinoma treated with radiotherapy	Diarrhoea and vomiting at time of PJI	No	No surgery	Benzyl penicillin 3 million units QDS and fusidic acid 500 mg TDS for two weeks, then oral amoxicillin 500 mg TDS and probenecid 500 mg QDS for four weeks	No recurrence at 19 months
Vogely HC, et al. [29]	1999	Hip	52	F	Late	Monomicrobial	N/A	N/A	No GI symptoms at time of PJI, acute cholecystitis six months before PJI	N/A	Two-stage exchange (gentamicin-containing cement)	IV penicillin and oral clindamycin	No recurrence at one and a half years
Pearle AD, et al. [30]	2003	Knee	82	M	Early	Monomicrobial	Negative	History of unknown bleeding dyscrasia	No	Yes	Two-stage exchange	IV penicillin for six weeks	No recurrence at six months
Lazzarini L, et al. [31]	2004	Hip	80	F	Early	Monomicrobial	N/A	No	No	N/A	DAIR	Penicillin and clindamycin for 30 days, then oral amoxicillin 1 g TDS for two weeks	No recurrence at 12 months
Baker JF, et al. [32]	2012	Hip	87	F	Early	Monomicrobial	N/A	No	No	Yes	DAIR	IV piperacillin/tazobactam and metronidazole for seven days, then oral metronidazole for four weeks	No recurrence at three months
Zafar Y, et al. [33]	2019	Knee	77	M	Late	Monomicrobial	Negative	Diffuse large B-cell lymphoma stage II-a in sigmoid colon treated with sigmoidectomy and chemotherapy	No GI symptoms at time of PJI, investigations revealed acute on chronic cholecystitis, colonoscopy revealed hyperplastic polyps and tubular adenoma	N/A	Irrigation and debridement with polyethylene exchange without replacement of the prosthesis	IV ertapenem 1 g OD and IV moxifloxacin 400 mg OD for six weeks and then oral moxifloxacin 400 mg OD for a total of 12 months	No recurrence at six months
Stroud G, Vandiver JW [9]	2020	Knee	64	M	Early	Monomicrobial	Negative	No	No	Yes	Irrigation and debridement followed by two-stage exchange (insertion of spacer with vancomycin and tobramycin)	IV clindamycin 900 mg TDS and IV metronidazole 500 mg TDS for six weeks, oral penicillin 500 mg QDS for one year as suppressive therapy	No recurrence at 12 months
Visse M, et al. [12]	2021	Hip	70	M	Early	Monomicrobial	Negative	No	N/A	N/A	Mobile implant removal	Clindamycin 600 mg TDS and rifampicin 600 mg BD for eight weeks, then metronidazole 500 mg TDS for four weeks	No recurrence, death due to ruptured aortic aneurysm
Visse M, et al. [12]	2021	Hip	86	M	Early	Polymicrobial (*Proteus vulgaris, Finegoldia magna, Peptoniphilus harei*)	Negative	Active cancer	N/A	N/A	Mobile implant removal	Co-trimoxazole, levofloxacin and metronidazole 500 mg TDS for four weeks, then clindamycin 600 mg TDS for eight weeks	No recurrence, death due to metastatic prostate cancer
Stokey P, et al. [10]	2023	Knee	80	M	Late	Monomicrobial	N/A	No	Developed acalculous cholecystitis shortly after PJI	N/A	Two-stage exchange	Oral metronidazole TDS and IV ceftriaxone OD for six weeks after first-stage exchange and oral co-amoxiclav for three months as suppressive therapy after second-stage exchange	No recurrence at one year
Dähler R, et al. [34]	2025	Hip	75	M	Early	Monomicrobial	Negative	No	No GI symptoms at time of PJI, CT scan showed diverticulosis	Yes	DAIR with exchange of removable components	Penicillin for two weeks, amoxicillin for 10 weeks, rifampicin added when drains removed	No recurrence at three months

Clinical Presentation

Among the 16 detailed cases, the majority were early-onset PJIs (69%; 11/16) occurring within three months of prosthesis implantation. Affected joints were limited to the hips (56%; 9/16) and the knees (44%; 7/16). Patients had a median age of 72 years. Late-onset infections, occurring more than three months after prosthesis implantation, were commonly associated with a distant source, with a biliary source identified in 80% (4/5) of those cases [10,25,29,33]. Cases with positive blood cultures were suggestive of haematogenous spread from biliary [26], gastrointestinal, or genitourinary sources [28]. Radiological evidence of gas formation in the joint or periarticular tissue was reported in six of 16 cases (38%).

Management and Outcomes

Management strategies varied, with most patients undergoing surgical intervention and only two cases being managed non-operatively [24,28]. Operative options included two-stage exchange (31%; 5/16), DAIR (25%; 4/16), mobile implant removal (25%; 4/16), and arthrodesis (6%; 1/16). Penicillin was the most frequently used antibiotic, administered in 75% (12/16) of cases. Metronidazole and clindamycin were each used in 31% (5/16) of cases. No recurrences were reported in 81% (13/16) of cases, with follow-up durations ranging from three months to two and a half years. One patient remained on oral suppressive antibiotic therapy for one year [9], whilst another developed a persistent discharging sinus [24]. Two patient deaths were reported, one due to an underlying malignancy and the other due to a ruptured aortic aneurysm [12].

*Clostridioides* *difficile*

*C. difficile* is a gram-positive, anaerobic, spore-forming bacillus widely recognised for causing antibiotic-associated diarrhoea and pseudomembranous colitis [35]. While extraintestinal infections are rare, isolated cases involving PJIs have been reported in the literature. These infections pose diagnostic and therapeutic challenges due to their atypical presentation, underrecognition, and limited guidance on optimal management. To date, only a small number of confirmed cases of *C. difficile* PJIs have been reported, typically involving monomicrobial infections in immunocompromised or comorbid patients. In this section, we summarise the clinical characteristics, diagnostic findings, and management approaches reported in the available literature on *C. difficile* PJIs (Table 2).

**Table 2:** Summary of Clostridioides difficile PJIs M: male, F: female, IV: intravenous, PJI: prosthetic joint infection, BD: twice daily, TDS: three times daily, N/A: data not available, Early infection: occurred within three months of surgery, Late infection: occurred more than three months after surgery, GI: gastrointestinal, HIV: human immunodeficiency virus, *C. difficile*: *Clostridioides difficile*

Author(s)	Year	Joint	Age	Sex	Early/Late infection	Type of infection	Blood cultures	Immunosuppressive conditions	Gastrointestinal symptoms	Radiological evidence of intra-articular or periarticular gas	Surgery	Antibiotics (after culture results)	Outcome
Achong DM, Oates E [36]	1994	Hip	31	F	Late	Monomicrobial	Negative	Functional asplenia on background of sickle cell anaemia	Loose bowel movements and stool culture positive for *C. difficile* at the time of PJI	N/A	Incision and drainage	IV metronidazole	Patient died weeks later (cardiorespiratory failure)
Pron B, et al. [37]	1995	Knee	16	M	Late	Monomicrobial	N/A	Radiotherapy and adjuvant chemotherapy for 14 months for osteosarcoma	No	N/A	Arthrotomy, prosthesis removal, followed by amputation	Amoxicillin and ornidazole for four months, then rifampicin added. After prosthesis removal, prophylactic lincomycin, IV penicillin G, ornidazole for three weeks.	No follow-up
McCarthy J, Stingemore N [38]	1999	Hip	83	F	Late	Monomicrobial	N/A	No	History of diarrhoea and *C. difficile* positive stool culture during perioperative period of primary arthroplasty, one year prior to PJI. Stool culture negative at time of PJI.	N/A	Hardware removal and planned for revision	Oral metronidazole	No follow-up
Ranganath S, Midturi JK [39]	2013	Shoulder	47	F	Early	Polymicrobial (Coagulase-negative *Staphylococcus*)	N/A	Alcoholic Hepatitis	History of diarrhoea at time of primary arthroplasty, three months prior to PJI. No GI symptoms at time of PJI.	No	Debridement, replacement of implant and hardware and antibiotic spacer insertion	IV vancomycin and oral metronidazole	No follow-up, discharged against medical advice
Curtis L, Lipp MJ [40]	2013	Knee	61	F	Early	Monomicrobial	N/A	No	No	N/A	Removal of hardware, antibiotic spacer placement, immobilisation of knee with cement blocks and artificial rods followed by above-knee amputation	IV piperacillin/tazobactam for four weeks, metronidazole for three weeks	Pain in amputation stump 31 months after amputation
Brassine L, et al. [41]	2014	Hip	61	M	Early	Monomicrobial	Positive for *C. difficile*	HIV	No	N/A	Debridement and implant retention	Oral metronidazole for 12 weeks	No recurrence at two years
Al-Tawfiq JA, Babiker MM [42]	2020	Hip	28	F	Early	Monomicrobial	Negative	Background of sickle cell anaemia, hydroxyurea previously paused due to pancytopaenia	No	N/A	Two-stage exchange	Oral co-amoxiclav and metronidazole for one year	No recurrence at three years
Karczewski D, et al. [43]	2021	Hip	20s	M	Early	Monomicrobial, developed secondary infection (*Staphylococcus epidermidis*)	N/A	Chemotherapy for acute lymphoblastic leukaemia	No	N/A	Abscess drainage, exchange of mobile parts, arthroplasty removal and girdlestone resection, multiple girdlestone revisions, spacer insertion, implantation of non-cemented total hip arthroplasty	Metronidazole for three years as suppressive therapy	No signs of relapse one month after reimplantation of total hip arthroplasty
Song Y, et al. [7]	2021	Hip	75	F	Late	Monomicrobial	N/A	No	No	No	Two-stage revision, removal of prosthesis and debridement, followed by cement spacer with vancomycin	IV metronidazole for two weeks and oral metronidazole 400 mg TDS for four weeks after each surgical stage	No recurrence at one year.
Amaya SL, et al. [44]	2021	Knee	79	M	Late	Monomicrobial	Negative	Rectal adenocarcinoma, permanent colostomy	*C. difficile* infection during postoperative period following surgery for periprosthetic fracture, one year before PJI. Stool culture negative at time of PJI	No	Two-stage replacement, prosthesis and periprosthetic plate removal, spacer placement with antibiotic-loaded cement, followed by spacer exchange and permanent prosthesis placement two months later	Oral metronidazole for six weeks, after spacer exchange IV teicoplanin and oral rifampicin for six weeks	Died three months later (unrelated cause, respiratory failure due to pneumonia)
Jordan H, et al. [45]	2025	Hip	76	M	Early	Monomicrobial	Negative	Alcohol excess	*C. difficile* infection with profuse watery diarrhoea five weeks prior to PJI	N/A	Multiple debridements with implant retention	IV vancomycin and IV metronidazole 500 mg TDS for two weeks, oral metronidazole for four weeks, then oral doxycycline 100 mg BD as chronic suppressive therapy	No recurrence at one year

Clinical Presentation

A total of 11 cases of *C. difficile* PJI were identified in the literature. The majority of affected joints were hips (64%; 7/11), followed by knees (27%; 3/11) and one shoulder (9%). Patients had a median age of 61 years. Most infections were early-onset PJIs (55%; 6/11), occurring within three months of surgery.

Gastrointestinal Symptoms

Gastrointestinal symptoms were reported in four cases (36%; 4/11), all attributed to *C. difficile *infection [36,38,44,45]. Two of these patients developed PJIs one year later, with negative stool cultures at the time of joint infection [38,44]. Three of the four cases reporting gastrointestinal symptoms were late-onset PJI presentations, accounting for 60% (3/5) of all late-onset PJIs.

Management and Outcomes

Metronidazole was used in 10 of the 11 cases. The most common surgical intervention was two-stage exchange, performed in 36% (4/11) of cases. Three patients (27%; 3/11) were managed with DAIR, all of whom received metronidazole. Of these three patients, two had no recurrence, one of whom remained on long-term suppressive therapy [45].

The outcomes of *C. difficile* PJIs suggest these infections can be severe and potentially life-threatening. Two patients underwent amputation, a 16-year-old with no report of follow-up [37] and a 61-year-old who experienced ongoing pain in the amputated stump [40]. One patient in their 20s required multiple revisions and eventually underwent reimplantation of their prosthesis after more than four years of management, with no signs of infection at one-month follow-up [43].

Other *Clostridium* species

Beyond *C. perfringens* and *C. difficile*, several other anaerobic *Clostridium* species have been reported as rare but clinically significant causes of PJI (Table 3). These include *C. septicum*, *C. cadaveris*, *C. bifermentans*, *C. ramosum*, and *C. disporicum*. Many of these species are recognised components of the human gastrointestinal microbiota, or environmental organisms found in soil, reflecting their capacity to act as opportunistic pathogens in susceptible hosts [46,47]. While uncommon, their isolation in PJI should raise suspicion for underlying systemic disease. Notably, *C. septicum* has a well-established association with occult colorectal malignancy [11].

**Table 3:** Summary of other Clostridium species PJIs M: male, F: female, IV: intravenous, PJI: prosthetic joint infection, OD: once daily, BD: twice daily, TDS: three times daily, N/A: data not available, Early infection: occurred within three months of surgery, Late infection: occurred more than three months after surgery, HIV: human immunodeficiency virus, ITU: intensive therapy unit, *C. difficile*: *Clostridioides difficile*

Author(s)	Year	Joint	Age	Sex	Early/Late Infection	Pathogen	Type of infection	Blood cultures	Immunosuppressive condition	Gastrointestinal	Radiological evidence of intra-articular or periarticular gas	Surgery	Antibiotics (after culture results)	Outcome
Morshed S, et al. [48]	2009	Hip	60	F	Late	Clostridium cadaveris	Monomicrobial	N/A	Chemotherapy and radiotherapy for metastatic breast carcinoma	N/A	Yes	Debridement, hydrogen peroxide lavage, open packing, repeat debridement, primary closure over drains, prosthesis retention	IV clindamycin 900 mg TDS for six weeks and oral clindamycin 150 mg BD as chronic suppressive therapy	No recurrence until death 18 months later
Burnell CDC, et al. [8]	2011	Knee	87	F	Late	Clostridium septicum	Monomicrobial	N/A	Lymph node involvement of caecal adenocarcinoma	Caecal adenocarcinoma	Yes	Irrigation and debridement followed by prosthesis removal and spacer insertion	IV cefazolin and metronidazole for six weeks after both surgeries	Died of underlying malignancy and metastatic disease to liver and adrenal glands seven months after presentation
Economedes DM, et al. [11]	2012	Knee	74	M	Early	Clostridium septicum	Polymicrobial (*Enterobacter*, coagulase negative *Staphylococcus*)	Positive for *Clostridium septicum*	Invasive adenocarcinoma with no nodal disease	Obstructive colon cancer at hepatic flexure and distal transverse colon	N/A	Irrigation, synovectomy debridement, implant retention and polyethylene exchange	IV penicillin G for six weeks	Reinfection at nine months, underwent two-stage revision, asymptomatic for 17 months
García-Jiménez A, et al. [49]	2016	Knee	62	F	Late	Clostridium ramosum	Monomicrobial	Negative	Rheumatoid arthritis on methotrexate and prednisolone	N/A	N/A	Prosthesis removal and cement spacer placement, followed by surgical debridement and cement spacer replacement followed by arthrodesis	Meropenem followed by oral metronidazole for six weeks. After arthrodesis, IV penicillin and clindamycin followed by oral metronidazole for total of three months	No recurrence at follow-up
McBride JA, et al. [50]	2017	Knee	70	M	Early	Clostridium disporicum	Monomicrobial	N/A	Metastatic renal cell carcinoma	*C. difficile* infection following total knee arthroplasty	N/A	Operated due to suspicion of necrotising fasciitis, no necrotic tissue present, prosthesis retained	Penicillin G and clindamycin, discharged on penicillin G 8 million units OD, eight weeks later oral metronidazole 750 mg BD added. At 10 weeks, penicillin stopped. At one month, metronidazole switched to amoxicillin 500 mg BD as chronic suppressive therapy	No recurrence at one year
Barrett LF, et al. [51]	2020	Knee	67	M	Late	Clostridium bifermentans	Monomicrobial	Negative	Substance abuse, was hospitalised with rib fractures, chest haematoma and bilateral lower lobe bronchopneumonia at time of PJI and admitted to ITU with septic shock	N/A	N/A	Washout and debridement with retention of prosthesis	IV ampicillin/sulbactam for two weeks, then oral co-amoxiclav for six months	No recurrence at three months
Moran M, et al. [52]	2023	Knee	61	M	Late	Clostridium bifermentans	Monomicrobial	Negative	HIV	N/A	No	Irrigation and debridement, haemovac drain insertion	IV vancomycin, IV cefepime, IV ampicillin/sulbactam	No follow-up

Clinical Presentation

A total of seven cases of PJI caused by *Clostridium* species, other than *C. perfringens* or *C. difficile*, were identified in the literature. These infections involved predominantly knee prostheses (86%; 6/7), with a single case affecting the hip [48]. Patients had a median age of 67 years. In contrast to PJIs caused by *C. perfringens* and *C. difficile*, most of these infections were late-onset (71%; 5/7), occurring more than three months after implantation. Notably, all affected patients had an underlying condition associated with immune suppression. In both *C. septicum *cases, identification of the pathogen prompted further investigations, which revealed previously undiagnosed colon adenocarcinomas [8,11].

Management and outcomes

All patients were managed surgically, with most cases involving implant retention (71%; 5/7). Of the two cases managed with implant removal [8,49], one resulted in arthrodesis [49]. The most commonly used antibiotics were penicillin (71%; 5/7), followed by metronidazole (43%; 3/7) and clindamycin (43%; 3/7). In two patients, lifelong suppressive therapy was initiated using clindamycin [48] and amoxicillin [50], respectively.

Outcomes were variable, with 57% (4/7) of cases completing treatment with no relapse at follow-up. One patient developed a reinfection nine months after the initial procedure and subsequently underwent a two-stage revision [11]. 

Discussion

Clostridial PJIs represent rare but clinically important causes of anaerobic implant infection. Across the published literature, *C. perfringens* accounted for the majority of reported cases (47%; 16/34), followed by *C. difficile* (32%; 11/34) and a smaller number of infections caused by other *Clostridium* species (21%; 7/34), including *C. septicum*, *C. bifermentans*, *C. cadaveris*, *C. ramosum*, and *C. disporicum*. The majority of affected patients were older adults with multiple comorbidities or immune suppression, reflecting the opportunistic nature of these organisms. Although uncommon, these infections are clinically important as their atypical presentation and anaerobic characteristics often delay diagnosis and appropriate therapy.

Clostridium perfringens

*C. perfringens* accounted for the largest proportion of clostridial PJIs in the literature and demonstrated a characteristic clinical pattern. Most infections presented early after arthroplasty, consistent with intra-operative contamination or colonisation of devitalised peri-prosthetic tissue. In contrast, late-onset cases were typically associated with a distant focus of infection, most frequently biliary [10,25,26,29,33] or gastrointestinal pathology [28], supporting the role of transient bacteraemia as a mechanism of prosthetic joint seeding. Radiographic evidence of gas formation, a hallmark of *C. perfringens* infection, was reported in multiple cases, supporting its value as a potential early diagnostic clue [6].

Clostridioides difficile

*C. difficile *PJIs were typically monomicrobial and occurred in patients with significant comorbidity. The predominance of immunocompromising comorbidities (8 of 11 patients) supports the hypothesis that *C. difficile* acts as an opportunistic pathogen in this setting. Furthermore, the absence of gastrointestinal symptoms in most cases suggests that haematogenous seeding from asymptomatic intestinal colonisation may be plausible. In line with these findings, several reports considered haematogenous spread the most likely source of infection [39,40,44]. Other authors proposed alternative mechanisms, including direct inoculation during surgery [38,45], iatrogenic introduction following joint aspiration [37], contamination through a fistula [36], or exposure during an anorectal bleeding episode [44]. Although only one case reported a positive blood culture [41], transient bacteraemia could explain haematogenous seeding not captured at the time of sampling.

Other Clostridium Species

Other *Clostridium* species constitute a very uncommon cause of PJI, but their identification is clinically significant due to their potential association with systemic disease. These cases presented predominantly as late-onset infections and occurred mainly in patients with underlying malignancy or immune suppression, suggesting an opportunistic pattern of infection. This pattern likely reflects their origin as gastrointestinal commensals, where disruption of mucosal integrity due to malignancy, inflammation, or infection may allow haematogenous translocation to the prosthetic joint. *C. septicum* was the most clinically significant of these species, its isolation being strongly associated with underlying malignancy. Due to this association, the literature emphasises the importance of species-level identification despite the additional laboratory time and cost [8]. Infection with this organism has been reported to coincide with malignancy in approximately 85% of cases, and may even represent the first indication of an occult malignancy [11].

Diagnosis

Diagnosis of clostridial PJIs is challenging as anaerobic organisms require specific culture conditions and prolonged incubation. Rapid specimen handling and the use of dedicated anaerobic culture techniques are essential to optimise diagnostic yield and minimise false-negative results [7]. In older reports, *Clostridium* species isolates were occasionally regarded as contaminants rather than true pathogens [36], likely contributing to historical underrecognition. However, contemporary evidence indicates that their isolation from joint aspirate or deep tissue should not be dismissed as contamination. Their presence should prompt careful clinical evaluation, as these organisms may represent true pathogens with important diagnostic and therapeutic implications. In addition to laboratory findings, imaging features may also provide useful clues. Gas within the joint or periarticular soft tissues, though not universally present, remains a useful radiological clue to anaerobic involvement [6]. Given the diagnostic challenges of anaerobic and toxin-producing species, the integration of rapid proteomic methods (e.g., MALDI-TOF mass spectrometry) and molecular techniques (e.g., 16S rRNA gene sequencing) may enhance species-level accuracy and early recognition in clostridial PJIs [16,17].

Management

Optimal management of clostridial PJIs requires prompt source control combined with targeted antimicrobial therapy [4]. Surgical strategies in reported cases included debridement with implant retention or exchange, with the exchange performed as either one-stage or two-stage procedures, depending on infection chronicity and patient stability. Arthrodesis or amputation was occasionally undertaken for uncontrolled infection. Notably, a small number of *C. perfringens* infections were successfully managed non-operatively with antibiotics alone [24,28], underscoring that carefully selected patients may respond to conservative treatment when surgical intervention is contraindicated.

The European Committee on Antimicrobial Susceptibility Testing (EUCAST) has published antibiotic minimum inhibitory concentration (MIC) breakpoints for *C. perfringens* and *C. difficile* [53]. For *C. perfringens*, antibiotics included penicillins, vancomycin, clindamycin, and metronidazole, among others, while for *C. difficile*, breakpoints are available for vancomycin, fidaxomicin, and metronidazole. Penicillin remains the cornerstone of therapy, retaining activity against most *Clostridium* species, and was the most frequently used antibiotic in cases not involving *C. difficile*. *C. difficile* cases were predominantly managed with metronidazole, often as monotherapy or in combination with other agents. The Pro-Implant Foundation Pocket Guide [54] currently represents the most widely adopted reference for PJI management in Europe and is considered the gold standard for clinical decision-making. Within its antimicrobial algorithms, anaerobic pathogens are discussed collectively, and recommended regimens include agents such as amoxicillin, clindamycin, and metronidazole. However, specific reference to *Clostridium* species or guidance on treatment duration for these organisms is absent. This omission underscores a persistent gap in contemporary practice guidelines, reflecting the rarity of clostridial PJIs and the paucity of data to inform pathogen-specific recommendations.

The randomised controlled trial by Li et al. in 2019 [19] supports consideration of oral step-down or outpatient parenteral antimicrobial therapy (OPAT) when bioavailable agents and adherence can be ensured. However, that study did not address the selection of specific antibiotics or pharmacokinetic factors such as bioavailability and tissue penetration. Antibiotic choice in PJI should balance biofilm activity, tissue penetration, and patient-specific factors [4], and incorporating outcome data linked to antibiotic selection and pharmacological properties would further inform optimal management of these uncommon infections. Across all reported clostridial PJIs, 71% (24/34) achieved infection control without recurrence, underscoring that these infections, though rare, are curable when recognised early and managed appropriately.

Limitations

Interpretation of our findings is limited by the small number and case-based nature of available reports. Most publications described individual cases, many of which were from earlier decades. Earlier reports pre-dating modern diagnostic criteria were included for completeness, and their findings should be interpreted in the context of historical laboratory and clinical practices. Details of antibiotic regimens, including dosing and route, were variably reported, and the rationale for surgical management was often not discussed. Before the introduction of molecular identification methods, occasional species misclassification may have occurred. Only English-language publications were included, which may have introduced language bias and led to omission of relevant cases reported in other languages, although no eligible papers were excluded on this basis. These factors should be considered when interpreting trends and drawing comparisons across cases.

## Conclusions

The isolation of a *Clostridium* species from joint aspirate or deep tissue warrants careful evaluation and should not be dismissed as contamination. Routine use of anaerobic culture media and extended incubation periods is vital in suspected PJIs, particularly when gas formation or tissue necrosis is present. Where local laboratories cannot perform anaerobic susceptibility testing, referral to a reference laboratory is advisable, since delays in obtaining results may postpone effective therapy. The isolation of *C. septicum* should prompt thorough malignancy screening. Early multidisciplinary management combining surgical source control with penicillin-based or other targeted antibiotic therapy offers the best chance of success; and in cases where implant retention is required, long-term suppressive therapy may be considered. Existing international guidelines for PJI management offer general principles but provide little organism-specific guidance for *Clostridium* species and no consensus on optimal treatment duration, highlighting a persistent gap in the literature. By consolidating all published clostridial PJI cases, this review defines their microbiological and clinical spectrum and offers practical insights into diagnosis and management of these uncommon but treatable infections. Given the diagnostic challenges associated with rare anaerobic organisms, the integration of advanced identification methods such as MALDI-TOF mass spectrometry and 16S rRNA gene sequencing would enhance species-level accuracy beyond conventional culture and improve recognition of clinically significant clostridial isolates. Future multicentre registries incorporating routine anaerobic surveillance and molecular characterisation will be essential to refine epidemiology, risk stratification and evidence-based management of clostridial PJIs.
